# 高流量湿化氧疗对单孔胸腔镜下肺叶切除术后患者的疗效分析

**DOI:** 10.3779/j.issn.1009-3419.2022.102.38

**Published:** 2022-09-20

**Authors:** 雪娟 朱, 潇梵 王, 星 金, 永华 桑, 文涛 杨, 勇兵 陈, 善州 段

**Affiliations:** 215004 苏州，苏州大学附属第二医院胸心外科 Department of Thoracic Surgery, the Second Affiliated Hospital of Soochow University, Suzhou 215004, China

**Keywords:** 经鼻高流量湿化氧疗, 无创辅助通气, 胸腔镜, 肺叶切除术, 低氧血症, High-flow nasal oxygen therapy, Noninvasive mechanical ventilation, Video-assisted thoracic surgery, Lobectomy, Hypoxemia

## Abstract

**背景与目的:**

部分胸外科患者术后拔除气管插管后并发低氧血症，常表现为Ⅰ型呼吸衰竭，改善低氧血症是促进患者术后康复的重要因素之一。本研究通过比较经鼻高流量湿化氧疗（high-flow nasal oxygen therapy, HFNO）、无创辅助通气（noninvasive mechanical ventilation, NIMV）及鼻导管吸氧（nasal oxygen breath, NOB）在单孔胸腔镜下肺叶切除术后并发低氧血症患者中的应用，探讨不同吸氧方式的优缺点，进一步探究HFNO在该类患者中的治疗效果。

**方法:**

选取2021年6月-2022年3月在苏州大学附属第二医院接受单孔胸腔镜下肺叶切除术后并发低氧血症的患者180例，随机分为三组（*n*=60），分别采用HFNO、NIMV及NOB治疗。观察各组患者使用前、使用1 h、使用6 h-12 h及停止使用后的动脉血气分析结果、患者舒适度以及并发症发生率，并使用SPSS 25.0软件进行统计分析，*P* < 0.05为差异有统计学意义。

**结果:**

对于单孔胸腔镜下肺叶切除术后并发低氧血症的患者，HFNO的治疗效果不逊于NIMV（*P*=0.333），相较于NOB，HFNO与NIMV均可较快提升患者的氧合指数（partial pressure of oxygen/fraction of inspiration O_2_, PaO_2_/FiO_2_）（*P* < 0.001）。除此之外，HFNO在患者使用舒适度、住院时间方面表现出绝对优势（*P* < 0.001, *P*=0.004），术后并发症较其他两组略有降低（*P*=0.232），但对于术后低氧血症并发动脉血二氧化碳分压（partial pressure of carbon dioxide, PaCO_2_）升高的患者，HFNO的治疗效果仍稍逊色于NIMV。

**结论:**

对于单孔胸腔镜下肺叶切除术后并发低氧血症的患者，HFNO可作为首选治疗方式；但对于术后低氧血症伴随PaCO_2_升高的患者，仍推荐使用NIMV改善氧合。

## Abstract (en)

**Background and objective:**

Patients who underwent lobectomy resection are prone to hypoxemia, and the vast majority present with type Ⅰ respiratory failure. Thus, improvement of hypoxemia is one of the most important factors to facilitate postoperative recovery of patients. In this study, the superiority-inferiority of different oxygen inhalation methods were compared with high-flow nasal oxygen therapy (HFNO), noninvasive mechanical ventilation (NIMV) and nasal oxygen breath (NOB) in patients with hypoxemia after single-port video-assisted thoracoscopic (VATS) lobectomy, and the clinical efficacy of HFNO in these patients was further investigated.

**Methods:**

A total of 180 patients from the Second Affiliated Hospital of Soochow University in China with hypoxemia who accepting single-port VATS lobectomy from June 2021 to March 2022 were randomly divided into three groups (*n*=60), which were treated with HFNO, NIMV and NOB, respectively. The results of arterial blood gas analysis, patient's comfort score and incidence of complications were observed before, 1 h, 6 h-12 h and after use. Statistical analyses were conducted using statistical program for social sciences 25.0 (SPSS 25.0), and *P* < 0.05 was considered as statistical significance.

**Results:**

For patients with hypoxemia after accepting single-port VATS lobectomy, HFNO was no less effective than NIMV (*P*=0.333), and both of whom could fast increase patients' partial pressure of oxygen/fraction of inspiration O_2_ (PaO_2_/FiO_2_) compared to NOB (*P* < 0.001). Besides, HFNO shows a great advantage in comfort degree and stay length (*P* < 0.001, *P*=0.004), and incidence of complications were slightly lower than other groups (*P*=0.232). But it is worthy to note that HFNO is still slightly less effective than NIMV in patients with postoperative hypoxemia accompanied by elevated partial pressure of carbon dioxide (PaCO_2_).

**Conclusion:**

For patients with hypoxemia who accepting single-port VATS lobectomy, HFNO can be used as the first choice. However, for patients with postoperative hypoxemia accompanied by elevated PaCO_2_, NIMV is still recommended to improve oxygenation.

肺部疾病的外科手术治疗经历多年的发展，现已囊括开胸手术、胸腔镜辅助手术（video-assisted thoracic surgery, VATS）和达芬奇机器人手术等多种形式^[1]^。近年来随着微创技术的研究深入，在三孔VATS的基础上进一步演化出单操作孔VATS及单孔VATS。单孔VATS于2004年首次被Rocco用于肺楔形切除^[2]^，并在近几年飞速发展，因单孔VATS将常规腔镜技术的观察孔及操作孔合并，极大程度上减轻手术创伤、降低手术风险，不仅有效缓解患者的术后疼痛程度，还有利于患者术后快速康复^[3]^，因此已经广泛应用于气胸、感染性疾病、良恶性肿瘤等的治疗。多项研究^[4, 5]^表明其在术后并发症、手术时间、淋巴结清扫、胸管引流量、住院时间和失血量等方面均优于多孔VATS。然而由于部分手术患者高龄、肥胖、肺功能欠佳、术后肺部渗出等原因，术后拔除气管插管后极易出现低氧血症，常表现为Ⅰ型呼吸衰竭，危重症患者需要二次气管插管甚至气管切开行机械辅助通气治疗。因此，如何克服胸外科肺叶切除术后严重低氧血症是影响患者术后康复的重要因素之一。目前临床治疗中常使用鼻导管吸氧（nasal oxygen breath, NOB）、无创辅助通气（noninvasive mechanical ventilation, NIMV）或经鼻导管高流量湿化氧疗（high-flow nasal oxygen therapy, HFNO）改善术后低氧血症。

NOB采用细长、顶端和侧面开孔的橡胶或塑料导管，将其插入鼻前庭使用，是目前国内各级医院普遍使用的吸氧方式，其优点在于依从性好、插入较浅、患者接受度高。NIMV是在无创呼吸机辅助下，通过面罩进气，减少自身呼吸做功，促进肺内氧合，是临床上常用的有效通气手段（2级证据，中度推荐）^[6]^，然而NIMV的使用需要大量资源支持，并且患者舒适度欠佳^[7, 8]^，在临床使用中存在一定局限。HFNO是通过将气流加温加湿并形成高速气流，经鼻进入气道后可产生平均2.7 cmH_2_O（1 cmH_2_O=0.098 kPa）的压力，形成呼气末正压通气，可促进氧合量，减轻呼吸功，并伴有最佳的热量和湿度。由于应用方便、患者耐受性良好及已被验证的临床益处^[9-11]^，现其已在临床中被广泛使用。本文通过比较经HFNO、NIMV及NOB在单孔胸腔镜下肺叶切除术后并发低氧血症患者的应用，探讨不同吸氧方式的优缺点，进一步探究HFNO在该类患者中的治疗效果。

## 1 资料与方法

### 1.1 一般资料

本研究已通过医院伦理委员会的批准，选取2021年6月-2022年3月在苏州大学附属第二医院接受单孔胸腔镜下肺叶切除术后并发低氧血症的患者，共计180例。依据术后出现低氧血症后首次血气分析中氧合指数（partial pressure of oxygen/fraction of inspiration O_2_, PaO_2_/FiO_2_），将患者按性别、年龄、吸烟史及基础疾病加以配对，以基本相同的三人为一组，共配成60组，然后将其随机分配于NIMV组、HFNO组及NOB组中，每组纳入60例。

### 1.2 纳入与排除标准

（1）纳入标准：①单孔胸腔镜下肺叶切除术后并发低氧血症，定义为PaO_2_/FiO_2_低于300 mmHg（1 mmHg=0.133 kPa）^[12]^或自主呼吸实验失败后动脉血氧饱和度（arterial oxygen saturation, SaO_2_）低于95%；②呼吸频率 > 20次/min，持续至少1 h，使用副呼吸肌或反常呼吸；③无严重的心脑血管疾病；④无头面部畸形或手术史，不影响HFNO及鼻导管吸氧操作；⑤无既往肺部手术史或严重胸部外伤史。（2）排除标准：阻塞性睡眠呼吸暂停综合征、谵妄、恶心呕吐、呼吸频率 < 14次/min、意识受损、血流动力学紊乱，患者及家属拒绝治疗者。

### 1.3 治疗方法

入组患者均在术前行肺功能检测，术中采用静脉吸入复合全身麻醉，双腔气管插管，健侧单肺通气，并接受单孔胸腔镜下肺叶切除术。术后前48 h常规使用单药抗感染、雾化吸入、平喘化痰、镇痛止血等对症处理。NIMV组使用Philips公司无创辅助通气系统，首次使用时间保持在2 h以上，待SaO_2_保持稳定后每2 h使用一次，若SaO_2_欠佳则延长使用时间，上机后根据患者临床表现、心电监测指标、血气分析结果视情况调整各项参数，使SaO_2_维持在98%以上。HFNO组使用Respircare公司HUMID-BH高流量湿化氧疗系统，初始吸氧浓度设置为50%，流量设置为40 L/min-50 L/min，温度37 ℃，保证连续使用，使SaO_2_维持在95%以上。NOB组使用鼻导管供氧，使SaO_2_维持在92%以上，所有受试者均保证术后至少12 h的使用时间。

治疗效果好转定义为停止使用后24 h内未表现出明显缺氧症状，如使用6 h-12 h后患者缺氧症状未见明显改善或血气PaO_2_/FiO_2_较前未见明显提升视为治疗无效，较前进行性下降视为治疗失败，并更改为给予气管插管或气管切开接呼吸机支持治疗。

### 1.4 检测指标

患者术后指脉氧监测SaO_2_低于95%时行血气分析，该结果定义为使用前血气分析结果。使用1 h、使用6 h-12 h的血气分析定义为：各组患者使用不同吸氧方式起1 h后、6 h-12 h时进行血气分析检测的结果。停止使用后血气分析结果定义为：①治疗好转患者停止使用后24 h、不吸氧状态下的血气分析结果；②治疗无效及治疗失败患者改予气管插管或气管切开操作时的血气分析结果。主要观测指标为患者使用前、使用1 h、使用6 h-12 h及停止使用后动脉血气分析的PaO_2_/FiO_2_，次要观测指标为动脉血二氧化碳分压（partial pressure of carbon dioxide, PaCO_2_），其他观测指标为患者使用前、使用1 h、使用6 h-12 h及停止使用后的酸碱度（potential of hydrogen, pH）、呼吸频率（respiratory rate, RR）、使用过程中的舒适度、皮肤损伤、术后漏气、肺不张、引流液是否 > 200 mL/d及术后住院时间。

### 1.5 统计学分析

研究结果使用SPSS 25.0软件进行统计分析，*P* < 0.05为差异有统计学意义。

## 2 结果

### 2.1 基线资料

NIMV组男性28例，女性32例，中位年龄67.5岁；HFNO组男性28例，女性32例，中位年龄68.5岁；NOB组男性25例，女性35例，中位年龄66.5岁。如表 1所示，三组受试者性别、年龄、吸烟史、既往基础疾病（高血压、肺气肿、心脏疾病）、手术切除位置、手术时长、每分钟最大通气量（maximal voluntary ventilation, MVV）及第一秒用力呼气量占所有呼气量的比例（forced expiratory volume in one second/forced vital capacity, FEV_1_/FVC）均无统计学差异（*P* > 0.05）。

**表 1:** 纳入患者的临床病理学特征 Clinicopathological characteristics of the included patients

Variables	NIMV group	HFNO group	NOB group	*P*
*n*	60	60	60	
Gender				0.817
Male	32	32	35	
Female	28	28	25	
Age (yr)	68 (57-78)	69 (56-80)	67 (53-75)	0.361
Smoking status				0.085
Non-smoker	37	41	48	
Current or former smoker	23	19	12	
Essential disease				0.164
Hypertension	13	19	11	
Pulmonary emphysema	17	13	9	
Heart disease	9	7	4	
Excision site				0.272
RU	23	15	17	
RM	4	5	5	
RL	6	14	15	
LU	14	11	6	
LL	13	15	17	
Duration of surgery				0.317
< 3 h	34	38	42	
≥3 h	26	22	18	
Preoperative pulmonary function				
MVV (MV/Pred %)	92.27 (86.13-97.69)	91.94 (86.21-97.96)	92.18 (86.02-97.80)	0.536
FEV_1_/FVC%	91 (83-97)	89 (83-97)	89 (83-96)	0.139
Baseline				
PaO_2_/FiO_2_	266 (233-297)	267 (251-284)	261 (248-283)	0.003
PaCO_2_	46.6 (39.7-50.3)	38.9 (38.1-39.6)	39.0 (38.1-39.6)	< 0.001
pH	7.39 (7.38-7.40)	7.39 (7.38-7.40)	7.39 (7.38-7.40)	0.428
RR	26 (18-33)	23 (22-24)	19 (14-23)	< 0.001
After 1 h				
PaO_2_/FiO_2_	270 (236-317)	304 (229-337)	283 (231-332)	0.270
PaCO_2_	44.7 (37.9-48.0)	38.6 (37.3-39.6)	38.5 (37.3-39.5)	< 0.001
pH	7.39 (7.38-7.40)	7.39 (7.38-7.40)	7.39 (7.38-7.40)	0.982
RR	26 (21-31)	22 (21-24)	21 (15-25)	< 0.001
After 6 h-12 h				
PaO_2_/FiO_2_	352 (239-402)	333 (230-381)	284 (231-337)	0.167
PaCO_2_	41.2 (36.6-49.7)	38.8 (37.6-39.8)	38.9 (37.6-39.8)	< 0.001
pH	7.41 (7.40-7.43)	7.42 (7.40-7.43)	7.42 (7.40-7.43)	0.855
RR	26 (20-30)	22 (20-24)	21 (16-26)	< 0.001
After use				
PaO_2_/FiO_2_	332 (242-383)	327 (206-376)	268 (201-336)	0.257
PaCO_2_	40.1 (36.6-50.4)	37.7 (36.2-39.5)	38.8 (37.4-39.8)	< 0.001
pH	7.40 (7.37-7.42)	7.40 (7.36-7.42)	7.39 (7.37-7.42)	0.506
RR	23 (19-31)	19 (15-26)	22 (16-35)	< 0.001
Comfort score				< 0.001
Good	9	39	45	
Acceptable	20	19	15	
Poor	31	2	0	
Skin injury				< 0.001
None	28	52	56	
Focal erythema	14	4	2	
Mild skin injury	10	3	2	
Skin ulcer	8	1	0	
Skin necrosis	0	0	0	
Postoperative complications				0.335
Pneumothorax	14	4	5	0.011
Pleural effusion > 200 mL/d	7	9	11	0.593
Pulmonary atelectasis	5	6	15	0.017
Stay length (d)	7 (5-12)	6 (3-13)	7 (3-13)	0.024
Outcome				< 0.001
Improvement	49	49	26	
No improvement	11	9	14	
Deterioration	0	2	20	
NIMV: noninvasive mechanical ventilation; HFNO: high-flow nasal oxygen therapy; NOB: nasal oxygen breath; RU: right upper; RM: right middle; RL: right lower; LU: left upper; LL: left lower; MVV: maximal voluntary ventilation; MV: measured value; Pred: predict; FEV_1_: forced expiratory volume in one second; FVC: forced vital capacity; pH: potential of hydrogen; RR: respiratory rate; SaO_2_: arterial oxygen saturation; PaO_2_: partial pressure of oxygen; FiO_2_: fraction of inspiration O_2_; PaCO_2_: partial pressure of carbon dioxide.				

### 2.2 不同吸氧方式对PaO_2_/FiO_2_的疗效比较

图 1A表现三组受试者使用前、使用1 h、使用6 h-12 h及停止使用后的PaO_2_/FiO_2_变化，使用6 h-12 h后患者氧合得到大幅改善，在停止使用后虽较前稍有回落，但仍趋于正常水平。如图 1B所示，三组患者使用前的PaO_2_/FiO_2_并无统计学差异，使用1 h后三组的PaO_2_/FiO_2_均较使用前提升，HFNO组提升最为明显（图 1C），较其他两组均有统计学差异（*P* < 0.05），NIMV组与NOB组提升程度相似（*P*=0.270）。HFNO组及NIMV组在使用6 h-12 h后PaO_2_/FiO_2_均高于NOB组（*P* < 0.05），NIMV组较HFNO组略高（图 1D），但并无统计学差异（*P*=0.241）。停止使用后HFNO组、NIMV组的PaO_2_/FiO_2_相似（*P*=0.134），且均高于NOB组（*P* < 0.05）（图 1E）。为进一步分析不同吸氧方式对使用前后PaO_2_/FiO_2_的提升水平，如图 1F所示，HFNO组与NIMV组对PaO_2_/FiO_2_的提升水平相似（*P*=0.079），且均优于NOB组（*P* < 0.05）。

**图 1:**
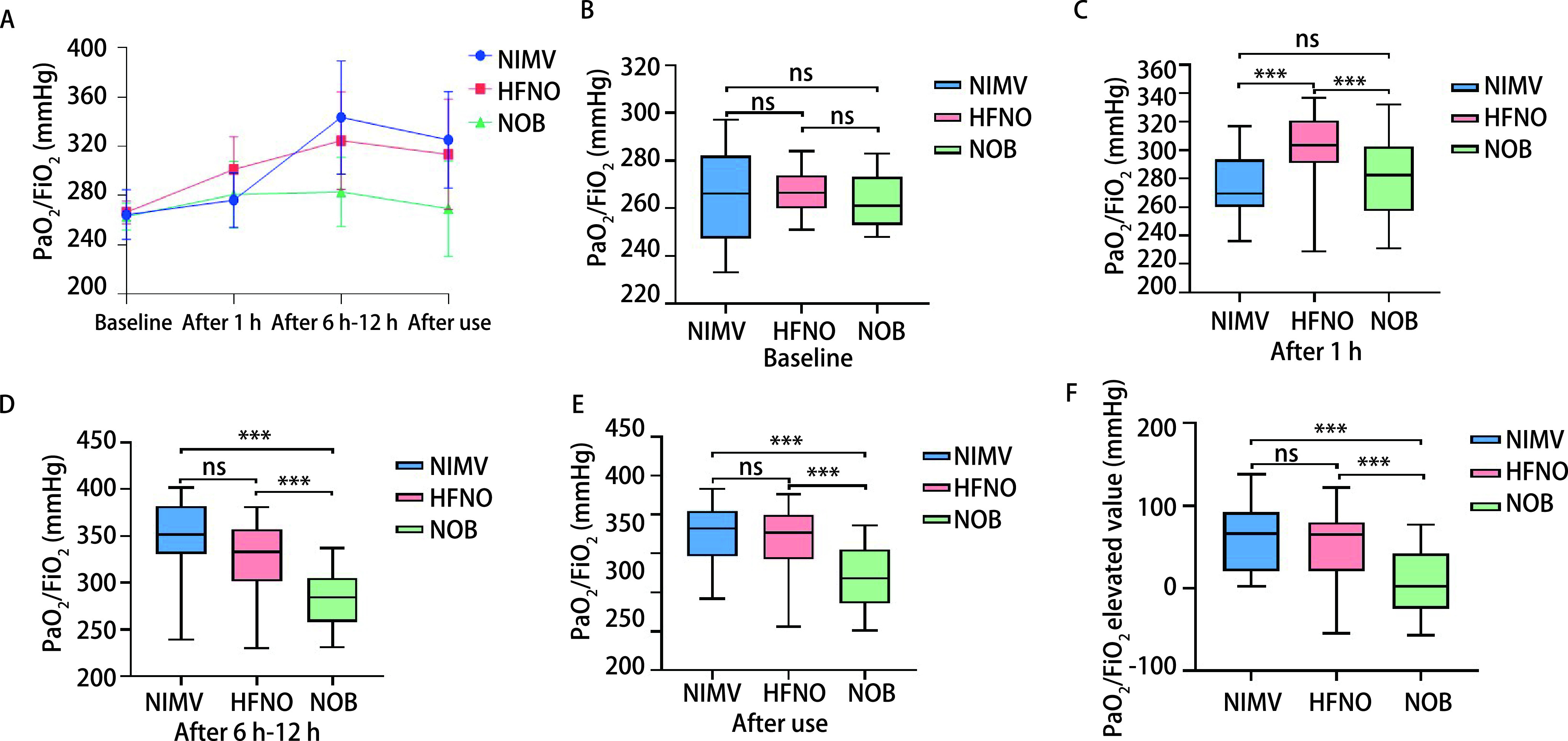
不同吸氧方式对PaO_2_/FiO_2_的影响。A：不同吸氧方式在使用各阶段PaO_2_/FiO_2_的变化值；B：HFNO组、NIMV组及NOB组开始使用前血气分析中PaO_2_/FiO_2_的比较；C：三组使用1 h后血气分析中PaO_2_/FiO_2_的比较；D：三组使用6 h-12 h后血气分析中PaO_2_/FiO_2_的比较；E：三组停止使用后血气分析中PaO_2_/FiO_2_的比较；F：三组使用前后血气分析中PaO_2_/FiO_2_提升值的比较。 The effects of different oxygen inhalation methods on PaO_2_/FiO_2_. A: Variation of PaO_2_/FiO_2_ at each stage by different oxygen inhalation methods; B: Comparison of PaO_2_/FiO_2_ in blood gas analysis among three groups of patients before use; C: Comparison of PaO_2_/FiO_2_ in blood gas analysis of three groups of patients following 1 h of use; D: Comparison of PaO_2_/FiO_2_ in blood gas analysis of three groups of patients after 6 h-12 h of use; E: Comparison of PaO_2_/FiO_2_ in blood gas analysis of three groups of patients after discontinuation; F: Comparison of PaO_2_/FiO_2_ elevated values in blood gas analysis of three groups of patients before and after use. ***P < 0.001; ns: not significant.

### 2.3 不同吸氧方式对PaCO_2_的疗效比较

通过分析三组不同吸氧方式各阶段血气结果中PaCO_2_的变化，如图 2A所示，HFNO组在使用6 h-12 h后PaCO_2_变化并不明显（*P*=0.138），但结束使用后PaCO_2_较前明显降低（*P* < 0.001），NIMV组PaCO_2_随使用时间呈下降趋势（*P* < 0.001）（图 2B），NOB组PaCO_2_波动并不明显（*P*=0.565）（图 2C）。图 2D分析不同吸氧方式使用前后PaCO_2_的降低程度，NIMV组的PaCO_2_降低程度优于HFNO组及NOB组（*P* < 0.001），且HFNO对PaCO_2_的改善情况与NOB组并无显著差异（*P*=0.996）。

**图 2:**
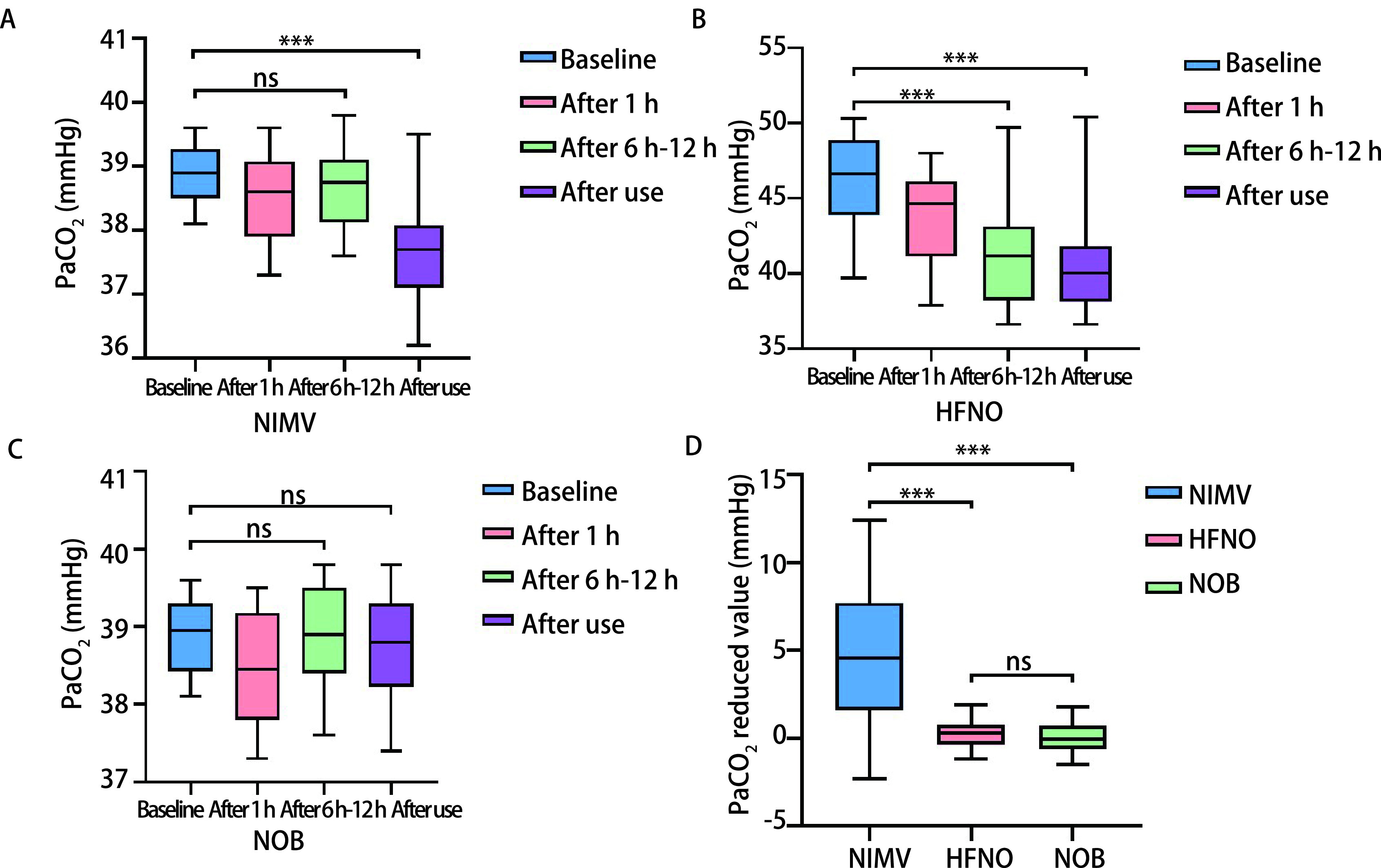
不同吸氧方式在各使用阶段PaCO_2_的变化值及对比。A：HFNO组在使用各阶段PaCO_2_的变化值；B：NIMV组在使用各阶段PaCO_2_的变化值；C：NOB组在使用各阶段PaCO_2_的变化值；D：三组使用前后血气分析中PaCO_2_/FiO_2_降低值的比较。 Alteration of PaCO_2_ under different oxygen inhalation methods and its comparison in each group. A: Fluctuation values of PaCO_2_ in the HFNO group at each stage avail; B: Fluctuation values of PaCO_2_ in the NIMV group at each stage avail; C: Fluctuation values of PaCO_2_ in the NOB group at each stage avail; D: Comparison of PaCO_2_ reduced values in blood gas analysis of three groups of patients before and after use. ***P < 0.001; ns: not significant.

### 2.4 不同吸氧方式对pH的疗效比较

如图 3A所示，三组吸氧方式对pH的影响均不明显，虽然在使用6 h-12 h后各组的pH值较使用前略有提升，但在各时间段内pH均稳定在标准范围内（pH标准值=7.35-7.45），组间及组内比较均证实其稳定性（*P* > 0.05）。

**图 3:**
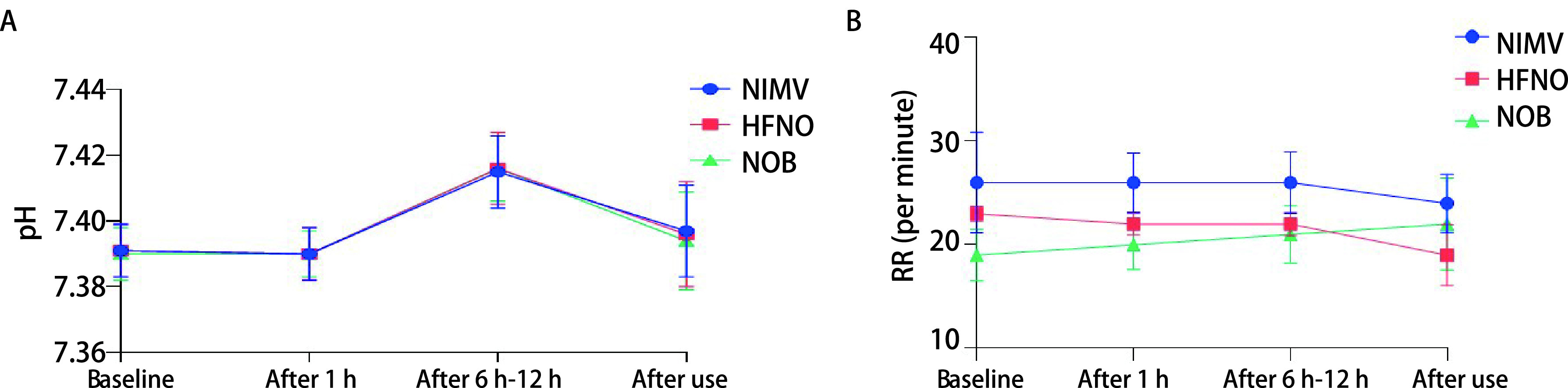
不同吸氧方式在使用各阶段pH（A）和RR（B）的变化值 Alteration of pH (A) and RR (B) under different oxygen inhalation methods

### 2.5 不同吸氧方式对RR的疗效比较

NIMV组、HFNO组在使用前后的RR均呈降低趋势（图 3B），而NOB组稍有增长，但各组RR的变化幅度均无统计学差异（*P* > 0.05）。

### 2.6 不同吸氧方式与术后并发症发生率的分析

HFNO组中仅有4例出现术后漏气、9例术后引流液 > 200 mL/d、6例肺不张，而NIMV组和NOB组的发生率分别为14例、7例、5例及5例、11例、15例（表 1）。进一步对各组行配对分析，如表 2所示，术后NIMV组漏气的发生率较HFNO组及NOB组高（*P*=0.011, *P*=0.024），HFNO组与NOB组漏气发生率并无显著差异（*P*=0.729）；HFNO组与NIMV组肺不张的发生率相似（*P*=0.752）且均低于NOB组（*P*=0.031, *P*=0.014），不同吸氧方式间对引流液的影响并不明显（*P*=0.591, *P*=0.624, *P*=0.306）。

**表 2:** 不同吸氧方式对术后并发症、舒适度评分、皮肤损伤、结局和住院时间的影响 Influences of different oxygen inhalation methods on postoperative complications, comfort score, skin injury, outcome and stay length

Variables	NIMV *vs* HFNO (*P* value)	HFNO *vs* NOB (*P* value)	NIMV *vs* NOB (*P* value)
Postoperative complications	0.232	0.166	0.849
Pneumothorax	0.011	0.729	0.024
Pleural effusion > 200 mL/d	0.591	0.624	0.306
Pulmonary atelectasis	0.752	0.031	0.014
Comfort score	< 0.001	0.235	< 0.001
Skin injury	< 0.001	0.569	< 0.001
Outcome	0.333	< 0.001	< 0.001
Stay length (d)	0.004	< 0.001	0.284

### 2.7 不同吸氧方式使用过程中舒适度及皮肤损伤的影响

术后NIMV组皮肤损伤的发生率为53.3%，其中严重皮肤损伤发生率为13.3%，31例自觉使用过程中出现严重不适、难以忍受的情况。HFNO组皮肤损伤的发生率为13.3%，其中严重皮肤损伤发生率为1.7%，2例重度不适，较NIMV组明显降低（*P* < 0.001）。NOB组皮肤损伤的发生率为6.7%，并未发现明显的皮肤损伤，HFNO组与NOB组间比较并无统计学差异（*P*=0.569）。对于不同吸氧方式，NIMV组患者的皮肤损伤与使用不适度均较其他两组高（*P* < 0.05），HFNO组无论是皮肤损伤还是使用舒适度，均与NOB组无明显差异（*P*=0.569, *P*=0.235）。

### 2.8 不同吸氧方式的治疗效果及其对住院时间的影响

经过治疗，NIMV组有49例好转，11例未见明显改善，0例治疗失败，HFNO组有49例好转，9例未见明显改善，2例治疗失败，NOB组有26例好转，14例未见明显改善，20例治疗失败，HFNO组与NIMV组的治疗成功率并无统计学差异（*P*=0.333），且两组的治疗成功率均优于NOB组（*P* < 0.001）。HFNO组较NIMV组及NOB组住院时间更短（*P*=0.004, *P* < 0.001），NIMV组与NOB组在住院时间方面并无明显差异（*P*=0.284）。

## 3 讨论

HFNO^[13]^作为一种新型呼吸支持技术，近年来在临床上得到广泛应用^[14]^，该设备主要包括空气-氧气混合器、活动加湿器、高流量鼻导管及连接的吸气回路^[15]^，为患者提供湿润的高流量气体（8 L/min-80 L/min），具有相对稳定的氧气浓度（21%-100%）及温度（31 ℃-37 ℃）^[16]^，可通过高流速气体产生呼气末正压通气（positive end-expiratory pressure, PEEP）样效应，产生一定水平的呼气末正压，降低气道阻力，减少呼吸做功^[17, 18]^，达到改善气体交换及部分通气功能。本研究与既往研究^[10, 11, 17]^得出一致结论，HFNO比NOB更能有效改善患者术后的氧合和舒适度。

低氧血症在接受单孔胸腔镜下肺叶切除术后的患者中很常见，通常采用NIMV治疗或预防^[7, 8, 19, 20]^。既往研究^[21-26]^提示，NIMV可改善肺叶切除术后并发低氧血症患者的预后，降低肺部并发症的发生及再插管的风险，但临床使用中，NIMV在患者选择方面仍存在一定的局限。NIMV所使用的呼吸面罩对密封性要求较高，从而导致患者鼻面部压迫、幽闭恐惧、舒适度差、耐受性低，直接影响治疗效果^[27]^。因此，HFNO被越来越多地应用于重症非手术期低氧血症患者中^[28]^。Ni等^[29]^的一项荟萃分析发现，与使用NOB的患者相比，使用HFNO的患者再插管率显著降低，本研究得出与其相似的结论，这一结果可能与HFNO的加热湿化功能有关。HFNO提供湿润的高流量气体具有保护黏膜、促进分泌物清除、减少气道塌陷、降低肺不张风险的优点，因此使用HFNO的患者较使用其他吸氧方式的患者呼气末肺容积更大，改善氧合的效果更佳。HFNO在使用过程中可产生PEEP样效应，而NIMV可给予患者外源性PEEP支持，因此，HFNO与NIMV对PaCO_2_的降低能力是否存在差异尚无统一定论^[30-33]^，在本研究中HFNO并未表现出对PaCO_2_降低的有利证据，其中可能存在手术麻醉的影响。

为进一步阐明HFNO的潜在临床意义，我们对HFNO和NIMV进行多方位的比较，在治疗效果、使用舒适度、患者配合度、术后并发症等各个层面，HFNO均表现出非劣性，因此，我们可以认为HFNO是胸外科肺叶切除术后并发低氧血症患者替代NIMV的良好选择。进一步研究得出，与NIMV相比，HFNO改善氧合的能力与其相似，同时拥有更高的舒适度及更低的皮肤损伤率，患者在使用过程中配合度高，术后住院时间进一步缩短，所以HFNO的治疗效果并不逊于NIMV。因NIMV在使用过程中需佩戴面罩，治疗期间无法进食，亦不利于交流与咳痰，但HFNO对其影响较小，故其方便快捷的使用方法更符合胸外科术后快速康复的治疗理念。

本研究表明，HFNO与NIMV的治疗效果均优于常规NOB。对于胸外科接受单孔胸腔镜下肺叶切除术后低氧血症的患者，HFNO可作为首选治疗方式，因其对PaO_2_/FiO_2_的改善效果不亚于NIMV，且HFNO的舒适度更高，可有效减少术后并发症的发生，加速术后恢复，缩短住院时间。但对于术后低氧血症伴随PaCO_2_升高的患者，仍推荐使用NIMV作为改善氧合的首选方式。
